# Small G protein Rac GTPases regulate the maintenance of glioblastoma stem-like cells *in vitro* and *in vivo*

**DOI:** 10.18632/oncotarget.14949

**Published:** 2017-02-01

**Authors:** Yun-Ju Lai, Jui-Cheng Tsai, Ying-Ting Tseng, Meng-Shih Wu, Wen-Shan Liu, Hoi-Ian Lam, Jei-Hwa Yu, Susan E. Nozell, Etty N. Benveniste

**Affiliations:** ^1^ Department of Life Science, National Taiwan Normal University, Taipei, Taiwan; ^2^ Department of Cell, Developmental, and Integrative Biology, University of Alabama at Birmingham, AL, USA; ^3^ Department of Biochemistry and Molecular Genetics, University of Alabama at Birmingham, AL, USA

**Keywords:** glioblastoma, Rac small G protein, glioblastoma-initiating cells, cancer stem cells, zebrafish xenotransplantation

## Abstract

Glioblastoma is the most common and aggressive malignant brain tumor in adults. The existence of glioblastoma stem cells (GSCs) or stem–like cells (stemloids) may account for its invasiveness and high recurrence. Rac proteins belong to the Rho small GTPase subfamily which regulates cell movement, proliferation, and survival. To investigate whether Rac proteins can serve as therapeutic targets for glioblastoma, especially for GSCs or stemloids, we examined the potential roles of Rac1, Rac2 and Rac3 on the properties of tumorspheres derived from glioblastoma cell lines. Tumorspheres are thought to be glioblastoma stem-like cells. We showed that Rac proteins promote the STAT3 and ERK activation and enhance cell proliferation and colony formation of glioblastoma stem-like cells. Knockdown of Rac proteins reduces the expression of GSC markers, such as CD133 and Sox2. The *in vivo* effects of Rac proteins in glioblastoma were further studied in zebrafish and in the mouse xenotransplantation model. Knocking-down Rac proteins abolished the angiogenesis effect induced by the injected tumorspheres in zebrafish model. In the CD133^+^-U373-tumorsphere xenotransplanted mouse model, suppression of Rac proteins decreased the incidence of tumor formation and inhibited the tumor growth. Moreover, knockdown of Rac proteins reduced the sphere forming efficiency of cells derived from these tumors. In conclusion, not only Rac1 but also Rac2 and 3 are important for glioblastoma tumorigenesis and can serve as the potential therapeutic targets against glioblastoma and its stem-like cells.

## INTRODUCTION

Glioblastoma multiforme (GBM) is the most common malignant primary brain tumor (classified as the grade IV astrocytoma), and one of the most aggressive cancers in humans. The median survival rate of GBM is 10 to 14 months after diagnosis. Aberrant regulations are noted during glioblastoma progression, such as transformation of developing neural stem cells [1, 2], and accumulation of genetic mutations in mature brain cells [3–5]. Glioblastoma has been grouped into four subtypes: classical, neural, proneural, and mesenchymal [6]. Each type is defined by specific sets of aberration and expression levels of regulatory genes. The classical subtype responds positively to aggressive therapies, while the prognosis of proneural and mesenchymal subtypes are much poorer [6, 7].

One of the main malignant characteristics of GBM is the high recurrent rate of tumors that are drug resistant. The existence of the glioblastoma stem cells (GSCs, or glioblastoma-initiating cells) may account for these properties. GSCs are heterogeneous cancer cells with stem cell-like properties. It has been proposed that GSCs are the subset among the tumor cells responsible for tumor initiation, invasiveness, resistance to treatments, and recurrence [2, 8]. Inhibition of GSCs may provide a strategy to target glioblastoma as an alternative to conventional therapy. However, some studies indicated that it is not necessary to have cancer stem cells to cause drug resistance, recurrence and metastasis [9–11]. A stemloid came from a proliferative cancer stem cell or an immortalized proliferating cell could produce the same outcome [12, 13]. These stem-like cells (stemloids) exist in most of tumor bulk and cancer cell lines. Hence, targeting these stemloids may also serve as an efficient strategy to treating cancer [11].

Rac proteins (ras-related C3 botulinum toxin substrate) are a subfamily of Rho small GTPases. The key function of this family is the regulation of actin cytoskeleton rearrangement, cell growth [14, 15], and the maintenance of stem cell during development [16]. In addition, recent studies have emphasized their role in tumor progression [17]. There are four members in the Rac family: Rac1, Rac2, Rac3 and RhoG [14]. Among them, Rac1 is the major member and has been studied most extensively [18–21]. Rac2 is expressed mainly in the hematopoietic cells but not in normal brain tissue [22]. It is critical for granule exocytosis in neutrophils [23], regulation of cyclooxygenase-2 expression [24], and maintenance of the cell morphology in macrophages [25]. Rac3 is expressed predominantly in brain with lower expression in other tissues [26]. It down-regulates autophagy [27] and functions as a pro-migratory co-activator of the nuclear receptor ERα [28]. All three members of Rac (Rac1-3) are involved in brain tumor progression. For instances, Rac1 enhances the survival of GBM cells through JNK pathway [29]. In a zebrafish transgenic model, Rac1 promotes active Akt1-induced gliomagenesis [30]. Moreover, inhibition of Rac1 activity in glioma cells with a small molecule inhibitor represses proliferation through G1 arrest and induces apoptosis [21]. From an integrated genomic analysis using The Cancer Genome Atlas (TCGA) database, Rac2 is highlighted by its over-expression in mesenchymal subtype, the most aggressive and the worst prognosis subtype among GBMs [6, 7]. Rac3 is mutated and over-expressed in brain tumor, and plays a role in aggressive glioma progression [31, 32].

Although Yoon et al. had reported that Rac1 is involved in the maintenance of stemness in GSCs *in vitro* [33], studies regarding the role of other Rac GTPase in GSCs and stemloids are limited. Here we demonstrated that not only Rac1 but also Rac2 and 3 are required for the growth, migration and invasion of glioblastoma stem-like cells. Moreover, Rac proteins promote the glioblastoma tumorsphere-induced angiogenesis in the zebrafish xenotransplantation model. Knockdown of Rac proteins also reduces the tumorigenesis in the mouse model *in vivo*. Therefore, targeting any of three Rac proteins is a potential strategy for GBM therapeutics.

## RESULTS

### Rac proteins promote the formation and proliferation of glioblastoma stem-like cells

Although a number of molecules have been identified to participate in glioblastoma progression [6, 34], there is little progress in effective targeting and controlling the relapse and invasion of GBM. There is increasing evidence that cancer stem cells or tumor-initiating cells may play a role in the relapse. To examine the effects of different Rac proteins in the formation and maintenance of glioblastoma-initiating cells, we first generated stable glioblastoma cell lines, U373-MG, U251-MG and U87-MG, that were infected with lentiviruses harboring green fluorescence protein (GFP), along with HA-tagged Rac cDNAs, or alternatively with shRNAs targeting specific Rac proteins mRNAs (shRacs). These cells were then cultured in neurosphere culture medium for a week to form tumorspheres. The mRNA expression levels of Rac1-3 in shRNA-transduced U251- and U373-tumorspheres were shown in Supplementary Figure 1A and 1B, respectively, while the protein levels in U373-tumorspheres were shown in Supplementary Figure 1C. The expression levels of HA-Rac1-3 were presented in Supplementary Figure 1D. To evaluate the infectivity of our lentiviruses, the infected cells expressing GFP were analyzed by Flow Cytometry and the percentage of positive cells were shown in Supplementary Figure 2A. In agreement, most tumorspheres were positive for GFP (Supplementary Figure 2B).

**Figure 1:**
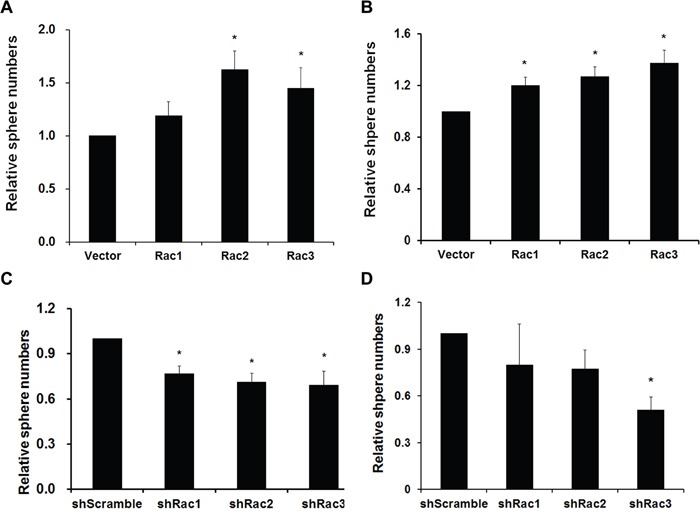
Rac proteins enhance the formation/maintenance of glioblastoma tumorspheres **A**. U251-MG and **B**. U87-MG cells stably expressing lentivector or different Rac proteins (Rac1-3), **C**. U251-MG and **D**. U373-MG cells expressing scrambled shRNA (shScramble) or specific shRNA targeting each Rac (shRac1-3) were counted, and 5×10^5^ cells/100 mm plate were cultured in neurobasal medium to form tumorspheres. After culture for one week, sphere colonies formed from single cells were fixed in 1% agarose gel and stained with 0.01% crystal violet. Colonies were imaged and quantified using the Gel Dock imager and Quantity One Software (BioRad). (p values are derived from Student's t-test. *: p<0.05, **: p<0.01).

**Figure 2:**
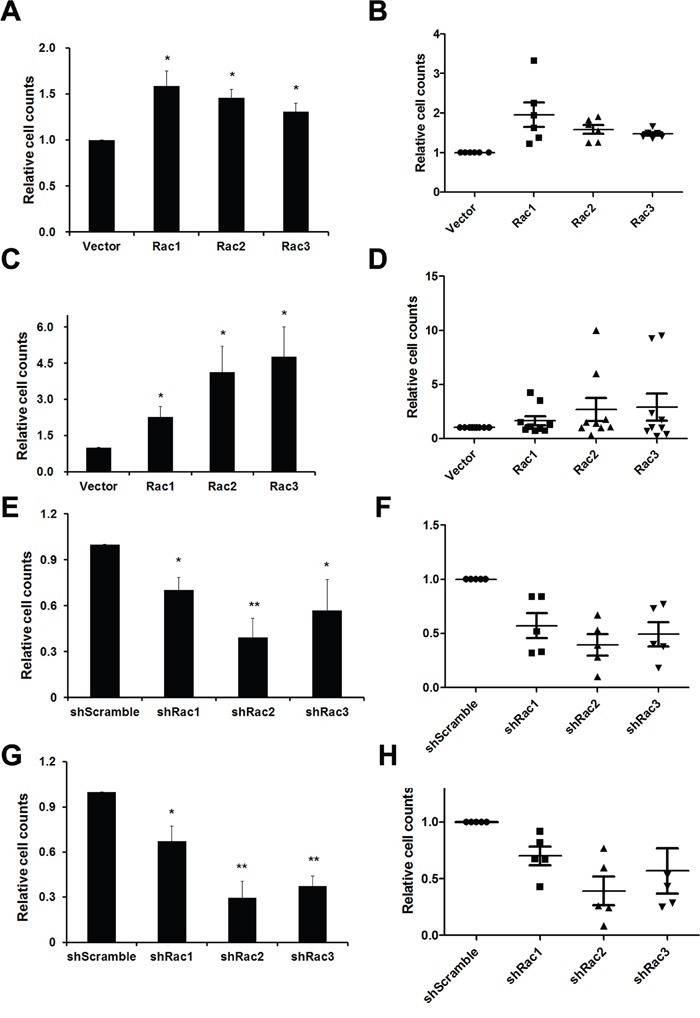
Rac proteins promote the cell proliferation of glioblastoma tumorpheres **A, B**. U87-MG and **C, D**. U251-MG cells stably expressing lentivector or different Rac proteins (Rac1-3) were counted and 5×10^5^ cells were cultured in neurobasal medium for a week to form tumorspheres. Single cell numbers of spheres were counted after each trypsinization when spheroid cells are needed to passage. Same experiments were also performed using U87-MG **E, F**. and U251-MG **G, H**. cells harbored control short hairpin RNA sequences and shRNA sequences targeting Rac1-3. A, C, E, G: Average cell counts from three independent experiments. (p values are derived from Student's t-test. *: p<0.05, **: p<0.01) B, D, F, H: Dot plots made by GraphPad Prism show each single counting during passages. Each dot represents each passage. (The lines represent the Q1, mean, and Q3 of quartile deviation.)

**Figure 3:**
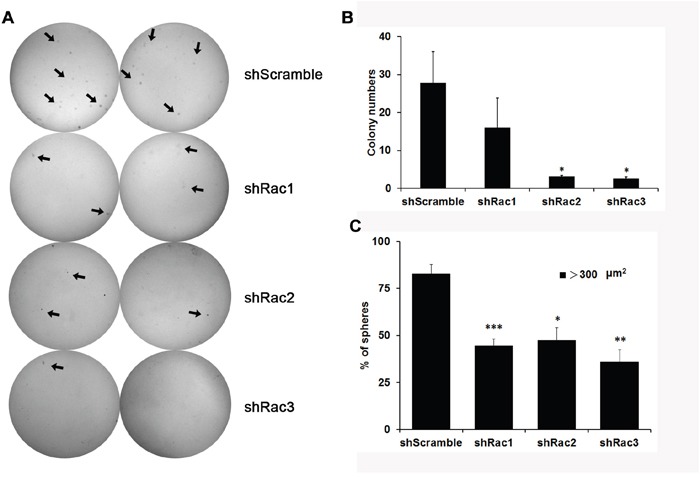
Knocking-down of Rac proteins reduces the colony formation of U251-tumorspheres **A**. U251-tumorsphere cells stably expressing scrambled shRNA (shScramble) or specific shRNA targeting each Rac (shRac1-3) were counted and 2,500 cells in 2x neurobasal medium were mixed with 0.7% agarose and were seeded in a well of 6-well plate. After culturing for three weeks, sphere colonies were stained with 0.01% crystal violet. Colonies were imaged by Las 3000 Image System and counted by Image J program. **B**. The quantitated results from at least three independent experiments (p values are derived from Student's t-test. *: p<0.05). **C**. The examination of the size of spheres. Ten thousand trypsinized U373-tumorsphere cells in 0.35% agarose containing medium were seeded in 12-well and cultured for a week. The tumorspheres formed in the gel were pictured by inverted fluorescence microscope and the area of the sphere was analyzed by Image J program.

The sphere formation ability was then determined by directly counting the sphere numbers. The results showed that, compared to the vector control, the number of tumorsphere was increased in U87-MG and U251-MG overexpressing Rac proteins (Figure 1A and 1B). In contrast, the sphere numbers decreased when the cells were transduced with shRacs compared to scramble control (Figure 1C–1D). To provide an objective and quantitative evaluation of cell proliferation, we counted the total cell numbers after trypsinizing the spheroids and found that, relative to the control, there were more cells when Rac proteins were ectopically expressed (Figure 2A–2B for U87-tumorspheres and C-D for U251-tumorspheres), and fewer cells in the presence of shRacs (Figure 2E–2F for U87 and G-H for U251-tumorspheres). The effects of altering Rac2 and Rac3 in U251 on tumorsphere formation and proliferation were higher than Rac1. This difference may be attributed to the higher expression of HA-Rac2 and 3 compared to HA-Rac1 (Supplementary Figure 1D). To verify that the effect of Racs on spheroid formation was not due to cell-cell aggregation, U251-tumorspheres harboring shRacs were examined by colony formation assay in the soft-agar with the neurosphere culture condition. The colonies were easily detected when cells harboring control shScramble, but not with cells expressing shRacs (Figure 3A, 3B). Furthermore, the sizes of tumorspheres-derived from U373-MG cells expressing shRacs were also smaller than those generated from scramble shRNA-control cells (Figure 3C). From these results, we conclude that the influence of Rac2 and Rac3 on the formation and proliferation of glioblastoma-stem like cells is no less than Rac1. These results make Rac2 and Rac3, in addition to Rac1, potential candidates for targeting GSCs.

### Rac proteins enhance the activation of STAT3 and ERK in glioblastoma cells, and the expression of stem cell marker CD133 and Sox2 in glioblastoma stem-like cells

**Figure 4:**
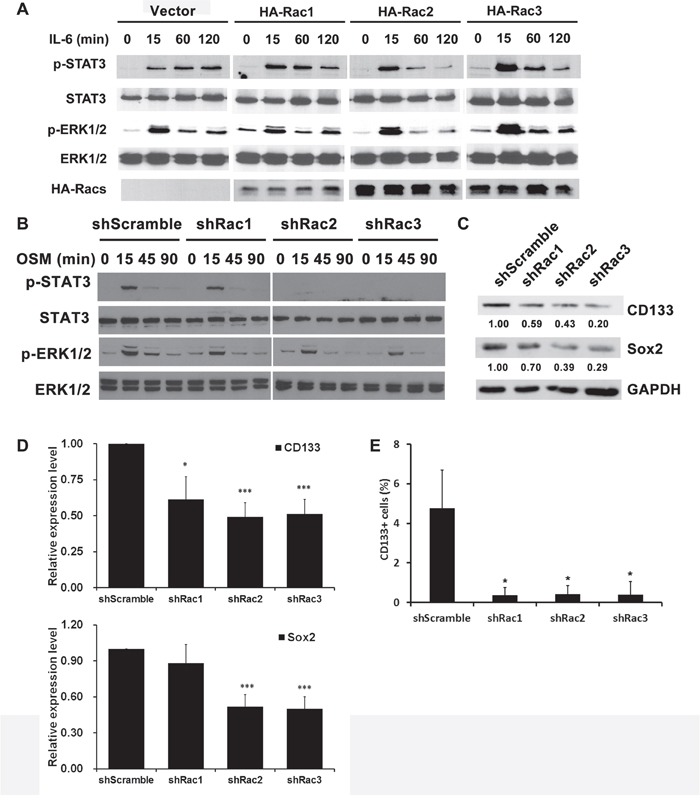
Rac proteins regulate the activation of STAT3 and ERK, and the expression of stemness markers in glioblastoma tumorspheres **A**. U87-MG cells transiently overexpressed HA-Rac proteins, or **B**. U251-MG cells stably expressing short-hairpin sequences to target specific Racs were starved for overnight and then treated with or without IL-6 (10 ng/mL) or oncostatin M (OSM, 10 ng/mL) for indicated time to induce the STAT3 and ERK activation. Protein lysates were prepared and subjected to immunoblot analysis with antibodies against phosphorylated STAT Tyr705, total STAT, phosphorylated ERK1/2 and total ERK1/2 antibodies. Ectopic expressed HA-Rac proteins were detected by anti-HA antibody. **C**. Lysates of U373-MG cells harboring control scramble sequences or shRacs were subjected to immunoblot analysis with antibodies against CD133 and Sox2 respectively. GAPDH detected by its specific antibody served as the loading control. **D**. The quantitative results for expression levels of CD133 and Sox2 from three independent Western blot analysis. **E**. U373 tumor spheroid cells stably expressing scramble shRNA or Rac1-3-targeting shRNA were treated with CoCl_2_ for 16 hrs. The surface CD133 expression was then detected by staining with PE-conjugated anti-CD133 antibody, and analyzed by flow cytometry. The results were normalized with IgG control staining. (*: p<0.05, t-test.)

We next examined the pathways involved in the proliferation and maintenance of GSCs. STAT3 and ERK are two major signaling pathways that regulate the growth of GSCs [35–37]. We found that, in response to IL-6, STAT3 and ERK were activated in U87-MG cells transiently expressing HA-tagged Rac1. Ectopic HA-Rac2 and Rac3 had a similar effect (Figure 4A). Conversely, knocking-down the expression of Rac1, Rac2 or Rac3 reduced the STAT3 and ERK activation in U251-tumorspheres (Figure 4B). Thus, not only Rac1, but also Rac2 and Rac3 had similar roles in regulating the growth of GSCs.

To elucidate the role of Rac proteins in supporting the maintenance of GSCs, we examined the expression levels of GSC markers, CD133 and Sox2. The results showed that knocking-down Rac expression in tumorspheres down-regulates the expression of CD133 and Sox2 (Figure 4C and 4D). Moreover, by flow cytometry, the CD133^+^-populations in Rac knockdown tumorspheres were significantly lower compared to the control tumorspheres (Supplementary Figure 3 and Figure 4E). These results suggest that Rac proteins also promote the formation and proliferation of glioblastoma stem-like cells by enhancing the expression of stemness genes.

### Rac proteins are required for glioblastoma stem-like cell migration and invasion

**Figure 5:**
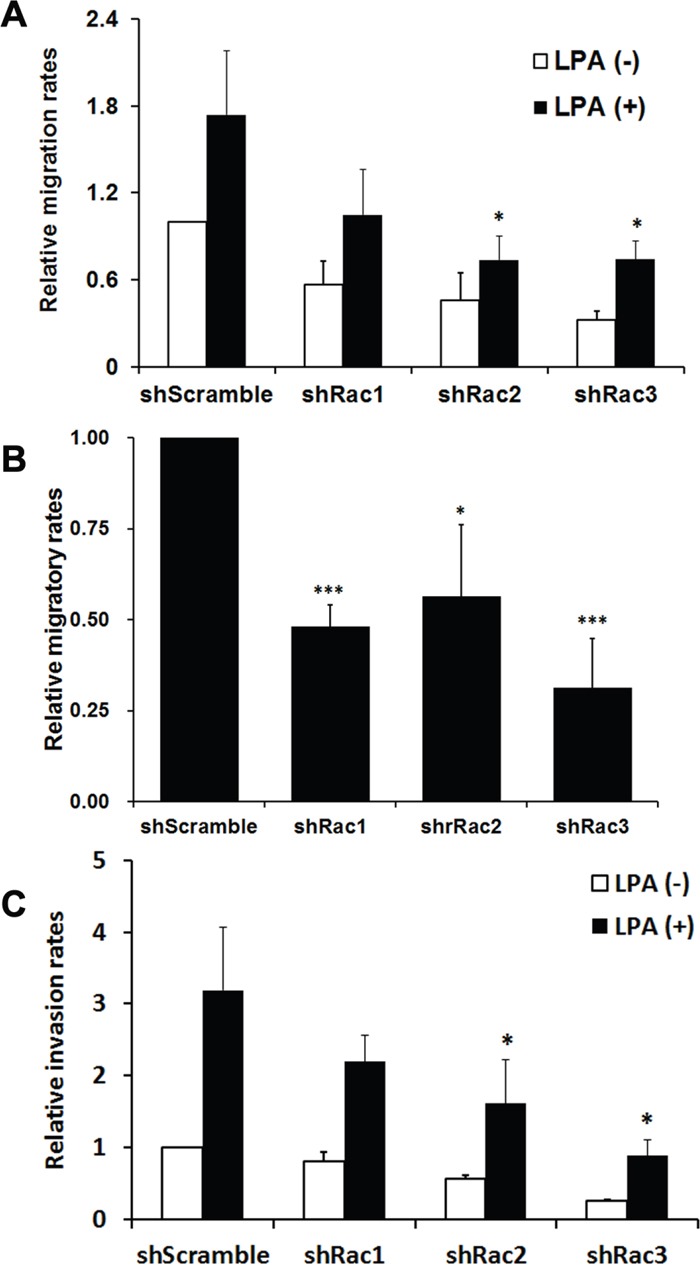
Rac proteins are important in LPA-induced migration and invasion of glioblastoma tumorspheres **A**. U251-MG or **B**. U87-MG cells stably expressing scrambled shRNA (shScramble) or specific shRNA targeting each Rac (shRac1-3) were cultured in neurobasal medium to form tumorspheres, and then subjected to Transwell cell migration assays. 10 μM LPA or 10% FBS was added to the lower chamber of Transwells that contain 3 μm-pore inserts, and cells were allowed to migrate for 6.5 hrs. The relative migration rate was defined as the fold-increase of migrated cells compared to migrated shScramble cells treated with vehicle. **C**. For the invasion assay, Transwell coated with 300 μg/ml Matrigel were used to examine the invasion ability of U251-siRac cells. Data shown are the mean ± SE of three independent experiments (*: p<0.05, Student's t-test).

The major function of Rac proteins is to regulate actin cytoskeleton, thereby controlling cell morphological changes and movement [15, 38]. To examine if Rac2 and Rac3, besides Rac1, also promote the migration and invasion of glioblastoma stem-like cells as they do in normal cells, we used the tumorspheroid cells derived from U251-MG that stably express shRNA or scramble shRNA to perform the cell migration assay using Transwell. The results showed that knocking-down each Rac by its specific shRNA significantly reduced the basal and LPA-induced tumorsphere migration (Figure 5A). Knocking-down Rac1 only caused about 25% decrease (statistically not significant), while knocking-down Rac2 and 3 caused more significant decreasing (about 50%) on relative cell migration. This difference might be due to the less efficient knockdown of Rac1 in U251-tumorsphere relative to Rac2 and 3 knockdown (Supplementary Figure 1A). Similarly, significant inhibition of cell migration was observed in Racs knockdown U87-tumorspheres (Figure 5B). To investigate the role of Rac proteins in the invasion ability of these tumorspheres, Transwell cell culture inserts coated with Matrigel® were used. Similar to the migration results, knockdown of Rac2 or Rac3 significantly reduced the invasion ability of U251-tumorsphere cells (Figure 5C). In summary, Rac2 and 3 along with Rac1 are required for cell migration and invasion in glioblastoma stem-like cells.

### The *in vivo* zebrafish xenotransplantation model

**Figure 6:**
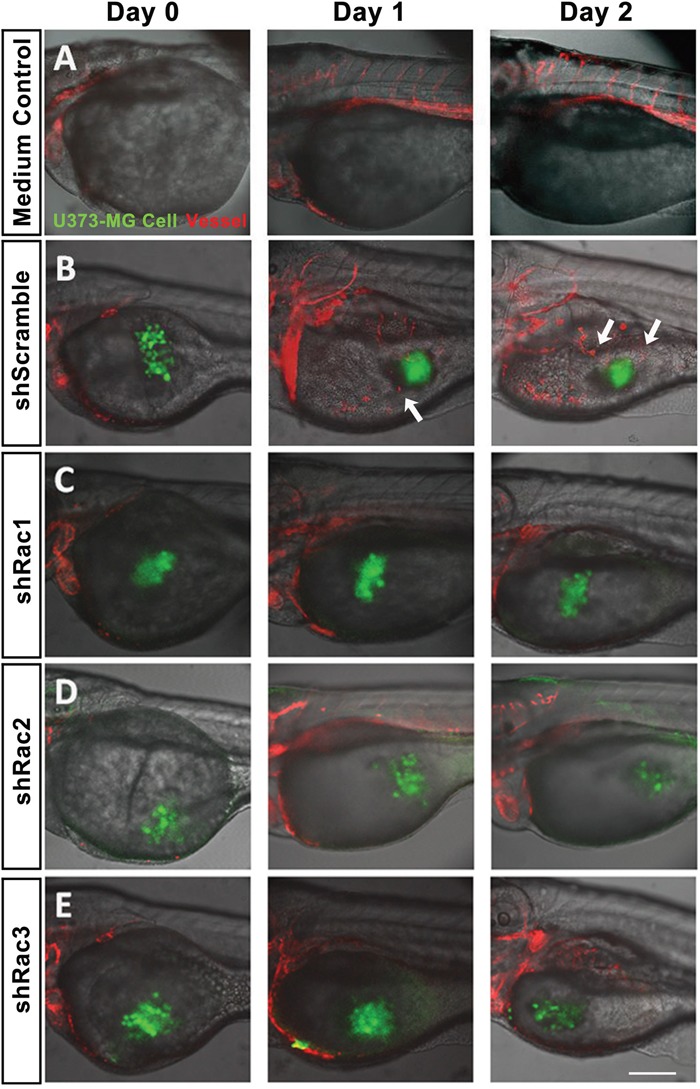
*In vivo* zebrafish xenotransplantation model of U373-MG tumorsphere cells with shRacs Tumorspheroid cells derived from U373-MG harboring control scramble sequences or shRacs and GFP expressing sequences were injected into the yolks of 2 dpf stage zebrafish *Tg(kdr:mcherry)* embryos. About 300~400 cells were injected for each embryo with 20% Matrigel in the sphere culture medium. Culture medium without cells was also injected into the yolk of embryos to serve as negative control. Confocal microscopy images were taken on the time indicated (TCS SP2, Leica). **A**. Inject with medium only. **B**. U373-shScramble cells. **C**. U373-shRac1 cells. **D**. U373-shRac2 cells. **E**. U373-shRac3 cells. Data shown were the representative from at least three independent experiments. Embryos injected number: n=20 for each group. GFP (green) represents glioblastoma spheroid cells, and mCherry (red) represents the vessels of the zebrafish embryos. Arrows show the angiogenesis revealing new vessels growing towards the injected tumor spheroid cells. Scale bar = 200 μm.

**Figure 7:**
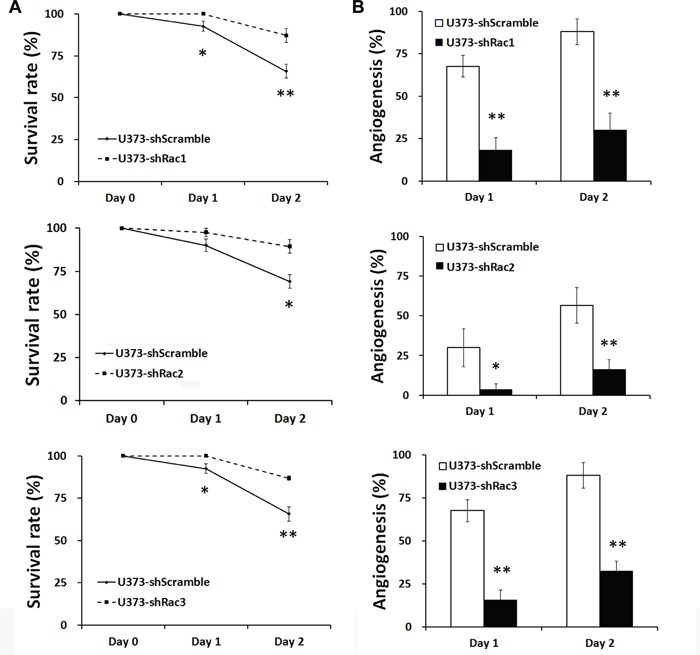
Glioblastoma tumor spheroid cells with reduced expression of Racs promote the survival of fish embryos and eliminate the angiogenesis induced by xenotransplanted tumor cells **A**. The statistical analysis for the survival of injected embryos in Figure 6. The survival rates were calculated by the number of survived fishes divided by total fishes injected with same cells. **B**. The statistical analysis for the angiogenesis of injected embryos in Figure 6. The embryo with vessel branches invading from main vessels into egg yolk were considered as angiogenesis. The ratios were obtained from the positive fishes divided by total fishes injected with the same cells.

**Figure 8:**
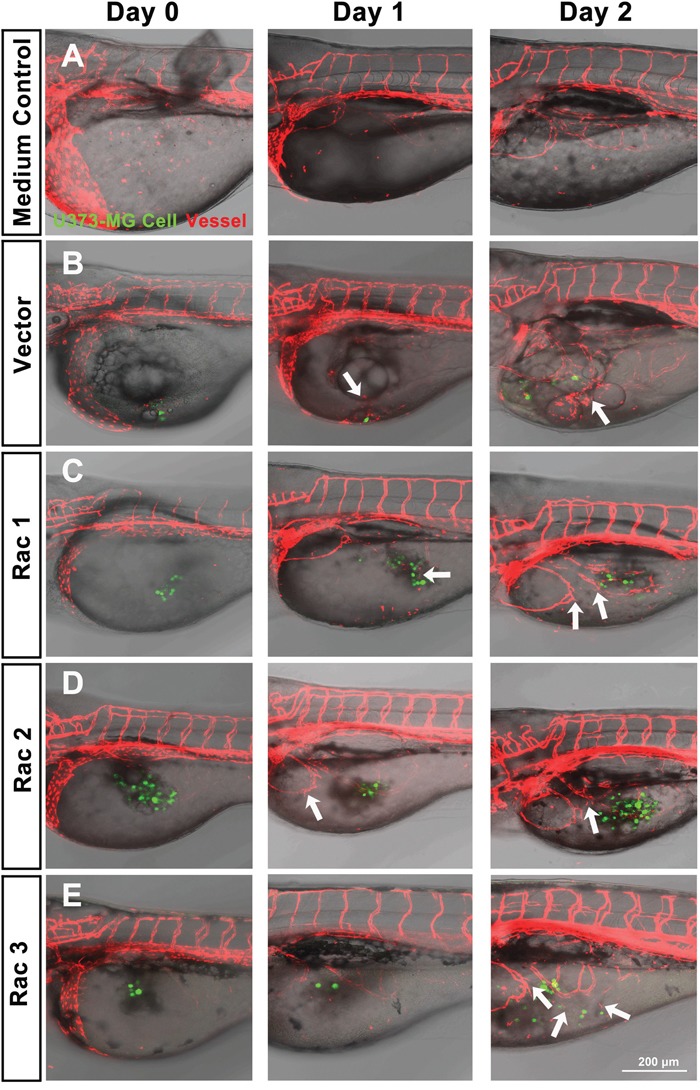
*In vivo* zebrafish xenotransplantation model of U373-MG tumorsphere cells with overexpressed Racs Same as described in Figure 6, tumorspheroid cells derived from U373-MG harboring control GFP or Rac cDNAs were injected into the yolks of 2 dpf stage zebrafish *Tg(kdr:mcherry)* embryos. Confocal microscopy images were taken on the time indicated (LSM880, ZEISS). **A**. Inject with medium only. **B**. U373-vector cells. **C**. U373-Rac1 cells. **D**. U373-Rac2 cells. **E**. U373-Rac3 cells. Data shown were the representative from at least three independent experiments. Embryos injected number: n=10~15 for each group. GFP (green) represents glioblastoma spheroid cells, and mCherry (red) represents the vessels of the zebrafish embryos. Arrows show the angiogenesis which the new vessel branches approaching the injected tumor spheroid cells. Scale bar = 200 μm.

**Figure 9:**
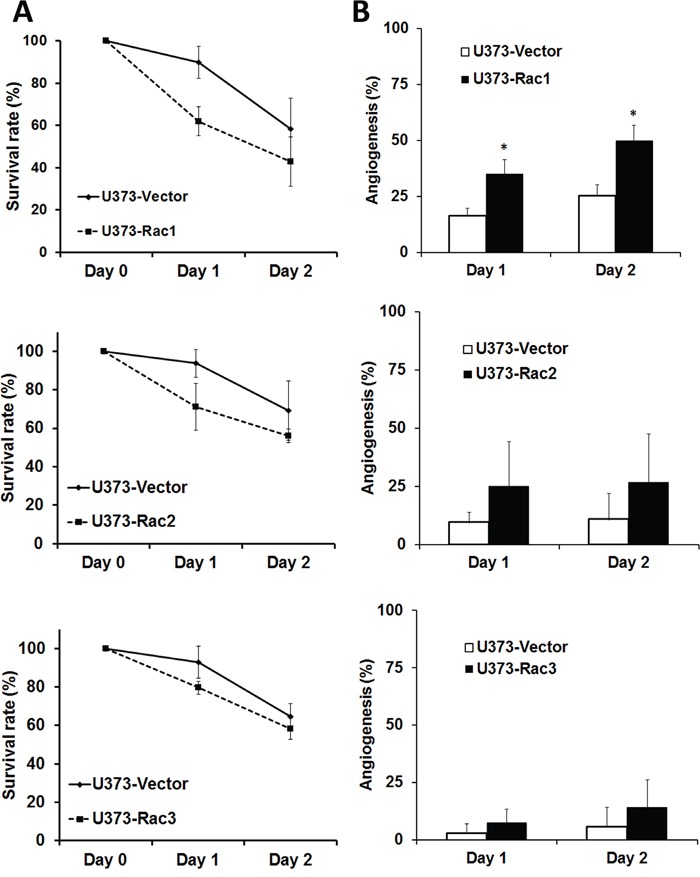
Glioblastoma tumor spheroid cells with overexpressed Racs reduce the survival of fish embryos and promote the angiogenesis induced by xenotransplanted tumor cells The statistical analysis for the **A**. survival and **B**. angiogenesis of injected embryos in Figure 8.

The zebrafish (*Danio rerio*) has served as an experimental vertebrate animal model for decades for several reasons: 1. Many embryos in each labor and a short embryo development period; 2. Ease of genetic manipulation and 3. High transparency for convenient live observation [39]. Although there were many xenotransplantation models of human cancer using zebrafish, most used cancer cell lines instead of cancer stem cells. To examine the effects of Rac proteins on glioblastoma progression *in vivo*, we took advantages of the zebrafish. We injected the GFP-labeled U373-tumorsphere cells into the yolks of zebrafish embryos (Figure 6). We observed higher survival rates of embryos bearing the tumorspheres derived from U373-MG cells with shRacs than the embryos with control scramble shRNA (Figure 7A). Instead of aggressive proliferation and migration, to our surprise, we observed a higher incidence of angiogenesis in embryos with control shScramble-expressing tumorsheres, but not in embryos with shRacs-expressing tumorspheres (Figure 6, 7B). In accordance, tumor spheroid cells overexpressing Racs increased the incidence of angiogenesis and reduced the survival rates (Figure 8, 9). The Racs overexpressing cells might have an aggressive proliferation since the xenografts shrank one day after injection but grew back the second day (Figure 8). These results support the notion that all three Rac proteins have a role in the aggressiveness and poor prognosis of glioblastoma.

### Knocking-down Rac proteins decreases the expression of VEGF and HIF-2α

**Figure 10:**
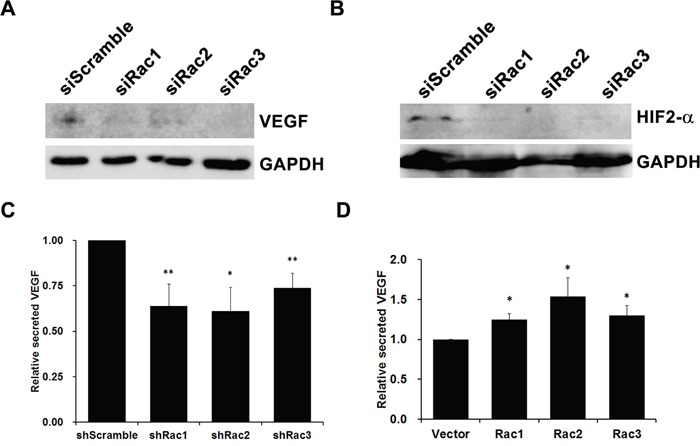
Rac proteins increase angiogenesis factors VEGF and HIF-2α expression in glioblastoma tumorspheres Lysates of U373-tumorspheres harboring control scramble sequences or shRacs were subjected to immunoblot analysis with antibodies against VEGF **A**. and HIF-2α **B**. GAPDH detected by its specific antibody served as loading controls. Secreted VEGF levels by **C**. shRacs or **D**. HA-Racs expressing U373-tumorspheres were detected by human VEGF ELISA Kit (PeproTech) from culture medium 6 hours or 48 hours after seeding. The VEGF levels were compared to the level secreted by shScramble or vector control cells (*: p<0.05, **: p<0.01, ***: p<0.005, t-test.).

We next examined the expression level of VEGF and HIF-2α, two major proteins that regulate angiogenesis in glioblastoma [40, 41]. The results show that U373-tumorspheres with Rac knockdown decreased the expression of HIF-2α and VEGF (Figure 10A, 10B). We also examine the level of secreted VEGF by ELISA assay and found that it was reduced from tumorspheres expressing shRacs (Figure 10C), whereas it was increased when tumorspheres overexpressed Racs (Figure 10D). Since HIF-2α is also a specific glioblastoma stem cell marker [41], this result strengthens the importance of Rac proteins in maintenance of GSCs.

### The *in vivo* mouse xenotransplantation model

**Figure 11:**
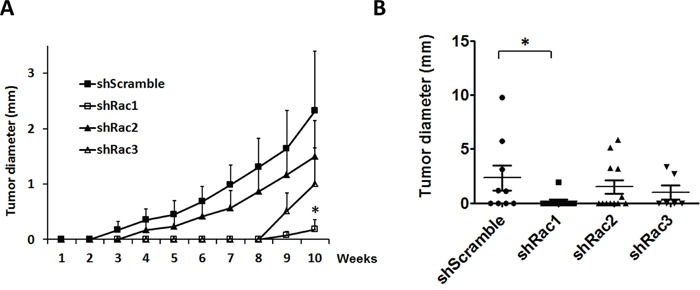
Rac protein knockdown suppresses the tumor progression in the mouse xenotransplantation model CD133^+^-tumorspheroid cells derived from U373-MG harboring scramble control or shRacs were injected into the NOD/SCID mouse flanks to generate the tumor. **A**. The average of tumor diameters in all groups. **B**. The diameters of each tumors were plotted by GraphPad. (The lines represent the Q1, mean, and Q3 of quartile deviation.).

To substantiate the role of Racs on the tumorigenesis *in vivo*, we generated a mouse xenotrasplantation model by injecting into NOD/SCID mice the CD133^+^-U373-tumorspheres expressing shScramble or shRacs. The average tumor size was smaller in mice with shRac-tumorspheres compared to shScramble control (Figure 11A, 11B). The incidence of tumor formation was also reduced in these mice (Table 1). To clarify that if the decrease of tumor incidence is due to a defect in adhesion ability of Rac knocked-down cells, we performed adhesion assays in U373-shRacs cells. There was no significant difference between control scramble shRNA and shRacs-expressing cells in their ability to adhere to collagen (Supplementary Figure 4). Moreover, we performed the tumorsphere formation efficiency (TFE) assay using the sub-cultured cells from these tumors to examine the stem cell populations persisted in the tumor. The results demonstrated that cells from tumors with shRacs are stemness-low compared to shScramble control (Table 1). Not surprisingly, we observed the highest inhibition of tumorigenesis in mice with shRac1. Although not significant, shRac2 or shRac3 also had similar inhibitory effects on tumor progression.

**Table 1:** Incidence of tumor formation and tumorsphere formation efficiency

	Tumor incidence		Spherogenic ability	
	Frequency (detected tumors/injected site)	%	Frequency (wells with colony/total well)	%
U373 shScramble	6 / 9	66.7	17 / 18	94.4
U373 shRac1	1 / 11	9.1	1 / 9	11.1
U373 siRac2	6 / 12	50.0	15 / 36	41.7
U373 siRac3	2 / 6	33.3	20 / 27	74.1

## DISCUSSION

The cause of drug resistance and cancer relapse have been attributed to the existence of GSCs or glioblastoma stemloids [11, 42]. Their identification also ignites a new search for therapeutic targets. The expressed markers and the regulation of these stem-like cells have been extensively investigated [42]. However, the identification of new target therapies is still limited. Here we report that Rac GTPase family as a potential target for treating glioblastoma stem-like cells. Targeting Rac GTPases by knocking down different Rac proteins in GBM-derived tumorsphere significantly reduces proliferation, colony formation, migration, invasion, and the expression of stem cell markers. Furthermore, repression of any of the three Rac proteins is sufficient to reduce tumorigenesis in our mouse model. Moreover, ectopic Rac proteins promote the angiogenesis in zebrafish xenotransplantation model, whereas shRacs reduce it.

Rac small GTPase subfamily composed of Rac1-3 has been studied primarily for their function in regulating cell motility. Therefore most studies have focused on their involvement in cancer cell migration, invasion and metastasis [43, 44]. Here we investigated the role of Rac GTPases in maintenance of GSC-like or stemloid cells. In addition to the well-studied Rac1, we also report the importance of Rac2 and Rac3 in the regulation of cancer stemloids. One evidence for the involvement of Rac in glioma progression is the dominant-active Rac1 (DARac1, Rac^G12V^) transgenic zebrafish model. DARac1 is not able to induce glioma, but promote the glioma progression in dominant-active Akt model [30]. A recent study further reported that Rac1 enhances the maintenance of stemness in GSCs *in vitro* [33]. Although the Rac2 is not expressed in normal nervous tissue [22, 45, 46], its expression is higher in glioblastoma compared to control tissue [47–49], and its overexpression is limited to the mesencymal subtype of glioblastoma [6]. In our studies, we detected Rac2 expression at the RNA level in U251- and U373-tumorspheres by RT-PCR, and further detected its protein expression in U373-tumorspheres. These results confirmed that the Rac2 is expressed in several glioblastoma samples. Furthermore, in our GSC-like stemloid cell model, Rac1, 2 and 3 each plays a critical role in maintenance of stemloid cells with regard to their ability of proliferation and colony formation (Figure 1 and 2), suggesting their involvement in glioma progression. To support our observations, the DNA copy number or mRNA expression of Rac proteins is higher in clinical glioblastoma than control tissues ([48, 49] and TCGA unpublished database).

The role of Rac GTPase in stem cells has been described in hematopoietic stem cells with respect to cell shape, adhesion, migration, mobilization and homing [50, 51]. However, except for the regulation of cell motility, as in glioma, Rac provides the survival signal in neuronal development, whereas Rho may induce apoptotic signal and promote the neuronal death [52]. The mechanisms mediated by Rac activation were previously investigated; it causes the activation of PI3K and PAK, which in turn activates Akt and ERK respectively [53]. In our studies, we observed a reduced ERK activation in glioma cell lines in which Racs were individually knocked down (Figure 4). However, whether it also affects the survival of tumorspheres needs to be further addressed. Rac1, 2 and 3 are also involved in BCR-ABL-mediated signaling pathway in HSC and leukemia stem cells [54]. The downstream signaling mediator of Rac is STAT5 in this case. However, we clearly detected reduced STAT3 activation in Rac down-regulated glioblastoma cells, but not STAT5 activation (data not shown.)

Although Rac1 has been reported to be one of the downstream effectors of VEGF signaling [55], down-regulation of Rac1 expression or activity also feeds back to reduce VEGF expression and VEGF-induced endothelial permeability and proliferation [56–58]. Furthermore, Rac1 also promotes tumor angiogenesis by inducing new vessel formation, and up-regulates the expression of HIF-1α and NF-κB, two major redox-dependent transcription factors induced by hypoxia environment [56, 59, 60]. In our zebrafish transplantation model, we also observed a role of not only Rac1, but also Rac2 and Rac3 in angiogenesis induced by glioblastoma tumorspheres. Upon expressing shRac1, shRac2, or ShRac3 in the tumorspheres, there was a reduction in angiogenesis, when compared to tumorspheres expressing shScramble. Conversely, ectopic expression of any of the three Racs promoted the angiogenesis in the xenotransplanted fish. In accordance, the secretion of VEGF is reduced in shRacs-expressing cells or increased in Rac overexpressing cells. Therefore, we conclude that promoting tumor angiogenesis is actively shared by all three Rac members. The application of Rac1 inhibitors in repressing angiogenesis has been reported in an *in vivo* mouse model [61]. Suppression of Rac protein expression therefore might serves as a new therapeutic strategy in VEGF blockade. Surprisingly, we did not observe the invasion phenomenon of these tumorspheres in our zebrafish model. This may be due to the slightly lower temperature for growing the cells (35°C instead of 37°C), the lower invasion ability of U373-MG derived-tumorspheres, or the short duration of these experiment due to the earlier death of the tumor-bearing fish embryos before post-fertilization day 7.

A correlation of Rac activation with clinical outcomes of malignant diseases has been suggested previously [62, 63]. In our zebrafish xenotransplantation model, we observed the better embryo survival after transplanting tumorspheres with shRacs relative to shScramble control. This observation suggests that level of Rac expression might serve as prognosis markers in glioblastoma. The possible cause of embryo death could be the aberrant angiogenesis which negatively correlated to the survival. Alternatively, the Rac expression levels of the xenogrfts might variably sensitize the embryos to death, especially when the embryos were kept in a warmer than usual temperature (35°C instead of 28°C). Aggressive growing may not be the reason for the death of embryos with shRNA expressing tumorspheres. However, it could have contributed the reduced survival of embryos with Rac overexpressing xenograft. We note that the latter xenograft shrank one day after injection but grew back the second day. This was not observed with xenografts with spheres expressing shRacs.

We further confirmed the tumorigenic ability of the expression of Racs in the mouse xenotransplantation model. The CD133^+^-U373-tumorspheres that represent GSCs were injected into immunocompromized mice and the Rac expression levels clearly correlated with tumor growth. Moveover, the tumorsphere formation efficiency assay demonstrates that the stem cell populations or stemloids in the tumors are reduced by shRacs.

Rac GTPases are also important in neuronal development. Three of the four members, Rac1, Rac3 and RhoG are expressed differentially in nervous system and have distinct function in neural development [45]. Neuronal polarity, extension and branching of neurites, and synapse formation are regulated by Rac via actin regulation. In addition, Rac has also been demonstrated to promote neuronal survival through the downstream effector p21-activated kinase (PAK) [64]. A recent study reported that Rac1 regulates the survival of cortical progenitors in the subventricular zone [65]. Moreover, Rac activation is required for the polarized outgrowth of protrusions in primary astrocytes during the initial phase of cell polarization [66]. However, this family of highly similar proteins may have overlapping roles in neuronal development, as the suppression of single Rac GTPase had little or no effect in normal neural system [45]. For instance, Rac3 knockout mice show no developmental defects [67]. Suppression of Rac1 expression in glioblastoma cell lines and human primary glioblastoma induce apoptosis but not in normal primary human adult astrocytes [29]. These findings reduce the concern for cytotoxicity in future application of targeting Racs in treating malignant brain diseases.

In summary, we have demonstrated the significance of Rac1-3 in promoting the maintenance of glioblastoma stem-like or stemloid cells, and they can serve as potential therapeutic targets against glioblastoma stemloids in the future. The differential requirements for Racs between normal neural cells and glioblastomas support this potential application.

## MATERIALS AND METHODS

### Plasmids

Rac1-3 cDNA were amplified from Racs 3xHA tagged clones (Missouri S&T cDNA Resource Center) and inserted into pIRES-GFP mammalian expression vector and pDL171-IRES-GFP lentiviral vectors. pSUPERIOR and pLVTHM vector (Addgene) was used to generate the shRNAs specific to target Rac1-3 (siRac1-3). The sequences targeting Rac1-3 were 5’- A AGC CTT CTT AAA GCC TTA-3’, 5’- AC TAC TCA GCC AAT GTG AT-3’ and 5’- GC GCC CAT GCA GGC CAT CA-3’, respectively. (pLVTHM vector also expresses GFP).

### Cell cultures and establishment of stable cell lines

Human glioblastoma cell lines U87-MG, U251-MG and U373-MG were maintained in Dulbecco's modified Eagle's medium (DMEM) (Gibco) with 10% fetal bovine serum (FBS) (Gibco) and 1% penicillin/streptomycin (Gibco) at 37°C, 5% CO_2_ incubator. The tumorspheres derived from these cells were maintained in NeuroBasal medium or DMEM//F12 with 1% B-27 without vitamin A (Gibco), 1% penicillin/streptomycin, 1% Amphotericin B, 10 ng/mL bFGF and 10 ng/mL EGF (Gibco) at 37°C, 5% CO_2_ incubator. To establish the Rac protein-overexpressing cells, lentiviruses with empty vector (pDL171-IRES-GFP, mock control) or harboring HA-Rac1-3 cDNA (pDL171-HA-Racs-IRES-GFP) were used to infect U87-MG, U251-MG and U373-MG cells. Lentiviruses pLVTHM harboring scramble shRNA or shRNA targeting Rac1-3 were used to generate the Rac knocking-down cells. The infectivity of lentivirus was over 80% in our observations and was also the case for other reports [68, 69]. Cells after lentiviral infection were used as the stable cell lines directly.

### Cell proliferation and colony formation assays

One hundred thousand cells were cultured in 60 mm plates and allowed to grow for a week to form tumorspheres. The numbers of spheres were then counted by plating 1 mL cell culture into the well of a 12-well plate and mixing with 1 mL 1% agarose. After solidification, 3% formaldehyde was used to fix the cells and 0.01% crystal violet was added to stain the cells. Spheres were imaged and quantified using the Gel Dock imager and Quantity One Software (BioRad). The sizes of spheres were then analyzed by Image J program (NIH).

For colony formation assay, 2,500 cells mixed in 0.35% agarose-containing culture medium were seeded on the top of 0.5% agarose medium in one well of 6-well plate. After four weeks of culturing, colonies were imaged by LAS3000 (GE) and the numbers and sizes of the colonies were analyzed by Image J program (NIH).

### Immunoblotting and RT-PCR

For immunoblotting, cells were lysed with 1xSDS lysis buffer (1% SDS and 60 mM Tris-HCl pH6.8) and sonicated for 20 seconds. Sixty μg of lysates were then applied to SDS-PAGE for immunoblot analysis. The target proteins were detected by their specific antibodies, including anti-HA (Santa Cruz), anti-Rac1 (BD Biosciences), anti-Rac2, anti-Rac3 (Abcam), anti-phospho-STAT3 Tyr705, anti-STAT3, anti-phospho-ERK1/2 Thr202/Tyr204, anti-ERK1/2, anti-Sox2 (Cell Signaling), anti-CD133 (Proteintech), anti-HIF-2α and anti-VEGF (Novas).

For RT-PCR, cells were subjected to RNA extraction using Trizol^®^ (Invitrogen) according to the manufacturer's instructions. Two μg of total RNA were reverse transcribed by GoScript™ kit (Promega) and then 2 μl of cDNA were used for PCR reaction by GoTaq^®^Green Master Mix (Promega). Forward primer sequence for detecting Rac1-3 is 5’- CCTGAGGTGCGGCACCACTG −3’, and reverse primer sequences for Rac1-3 are 5’- GCAGGCATTTTCTCTTCC-3’, 5’-GGCTGCAGGC GCGCTTCTG-3’, and 5’-CGGTGCACTTCTTCCCC GG-3’, respectively.

### Transwell migration and invasion assays

The Transwell migration assay was conducted as described previously [70]. In brief, 1×10^5^ cells trypsinized from U251 or U87-tumorsphere were re-suspended in 1% BSA containing serum free medium and seeded in a transwell of 12-well plate. Medium containing 10 μM LPA or 10% FBS was added in the lower chamber to induce cell migration. After 6.5 hours of incubation, the cells remaining inside the transwell were removed by cotton tips and the cells migrating to the other side of transwell were fixed with 3% formaldehyde and stained with 5 mg/mL crystal violet. The stained cells were then counted under inverted microscope (DMI3000, Leica). For invasion assay, 300 μg/ml Matrigel were used to coat the inner side of transwell and other steps were same as migration assay.

### Flow cytometry and enzyme-linked immunosorbent assay (ELISA)

Five hundred thousand trypsinized U373-spheroid cells were incubated with 0.3 mM CoCl_2_ for 24 hours and then stained with PE-conjugated anti-CD133 antibody (Miltenyi Biotec) for 2 hours to detect CD133^+^ cells. CoCl_2_ treatment was used to enhance the expression of CD133. The stained cells were analyzed by flow cytometry (Cell Quest) and FlowJo program. The same number of cells incubated with PE-conjugated IgG served as negative control.

For ELISA, 2 × 10^5^ U373-spheroid cells were seeded in one well of 6-well plates and incubated in normal culture condition for 6 or 48 hours, and the culture medium was collected to detect secreted VEGF by Human VEGF ELISA Kit (PeproTech).

### The zebrafish xenotransplantation model

*Tg(kdr:mCherry)* strain of zebrafish were kept under standard laboratory conditions of 28°C on a 14 hr light/10 hr dark photoperiod in aerated tap water. Embryos were kept under the same condition in distilled water with 0.5 mM NaCl, 0.2 mM MgSO_4_, 0.16 mM KH_2_PO_4_ and K_2_HPO_4_. All animal experimental procedures were approved by the Institutional Animal Care and Use Committee (IACUC) of National Taiwan Normal University (Approval Number 102032). Two days postfertilization (dpf) embryos were used for xenotransplantation. To avoid the fluorescent artefacts of the fish pigments, 0.2 mM PTU (1-phenyl 2-thiourea, Sigma) were added into culture water 1 dpf to inhibit the pigmentation.

The needles for injection were prepared from glass pipettes (Drummond Scientific) by a micropipette puller (kindly provided by Dr. Yung-Shu Kuan, National Taiwan University) with the following settings: pressure: 500; heat: 560; pull: 100; velocity: 50; delay time: 200. U373-MG cells harboring shScramble shRNA, shRacs, or Rac1-3 cDNA were prepared in 2.5×10^4^ cells/μL suspension with 20% Matrigel (BD). Eighteen nL of cell suspension (about 450 cells) were injected into the egg yolk of the embryo by an oil-driven pressure injector (Nanoject II) (Drummond Scientific). The immune system of zebrafish is not fully developed until 28 dpf, at which time, the immune responses would preclude the xenotransplantation of human cells [71]. After injection, embryos were kept in 31°C for an hour and then the incubator temperature were reset to 35°C for the rest of the experiment. Successfully injected embryos were observed by the inverted fluorescence microscope (DMI3000, Leica) and photographed by the confocal microscope (TCS SP2, Leica and LSM880, ZEISS) for the following three days. The embryos were anesthetized with 0.02% Tricaine (Sigma) in culture water during the injection and each microscopy processing. The embryo can survive un-feed for 6 to 7 days (7 dpf). The embryo with vessel branches invading from main vessels into egg yolk were considered as angiogenesis. The survival rate were calculated by the number of survived fishes divided by total fishes injected with same cells on 1 or 2 day-post-injection.

### Mouse xenotransplantation model

The animal protocol was approved by the Institutional Animal Care and Use Committee (IACUC) of National Taiwan Normal University (Approval Number 102025). Three weeks old female non-obese diabetic/severe combined immunodeficiency (NOD/SCID, NOD_CB17-*Prkdc*^scid^/JNarl) mice were obtained from National Laboratory Animal Center (NLAC, Taiwan) and maintained in cages with photoperiod (12L: 12D) in IVC system with food and water. One week later, 5×10^3^ trypsinized CD133^+^-U373-spheroid cells with shScramble or shRacs were re-suspended in 80 μL DMEN/F-12 containing B-27, EGF and bFGF, mixed with 20 μL Matrigel (356231, BD) and injected subcutaneously into one or both sides of the mouse flank. The tumor size was measured and recorded every other day.

### Primary cultures of xenografted tumor cells

After sacrifice the mice, tumors were dissected out and digested with 0.25% trypsin solution for 15 minutes. After neutralization with trypsin inhibitor and re-suspension in tumorsphere medium, sieved single cells were counted and 50 cells were seeded in 96-well and cultured for three weeks for tumorsphere formation efficiency assay. The efficiency was determined by counting the ratio of wells with successfully-formed spheres to the total wells with cell seeded.

### Statistical analysis

Student's *t*-tests were used in comparison between groups. All data were presented as mean ± standard error except for dot plots derived from GraphPad Prism program in Figures 2 and 11, that presented data recorded with Q1, mean and Q3 of quartile deviation.

### Grant Support

National Science Council; Grant number: NSC-100-2320-B-003-001-MY2.

Ministry of Science and Technology; Grant numbers: MOST 103-2320-B-003 −004, MOST 104-2320-B-003-010.

National Taiwan Normal University; Grant numbers: NTNU-103T3040B03, NTNU-104T3040D3.

## SUPPLEMENTARY MATERIALS FIGURES


