# Supplementary material for: Small G protein Rac GTPases regulate the maintenance of glioblastoma stem-like cells in vitro and in vivo
Source: Oncotarget. 2017 Feb 1;8(11):18031–49. doi: 10.18632/oncotarget.14949 (PMC5392305; doi:10.18632/oncotarget.14949)
Supplement: Supplementary file 1 [file oncotarget-08-18031-s001.pdf]

Small G protein Rac GTPases regulate the maintenance of glioblastoma stem-like cells *in vitro* and *in vivo*

SUPPLEMENTARY FIGURES

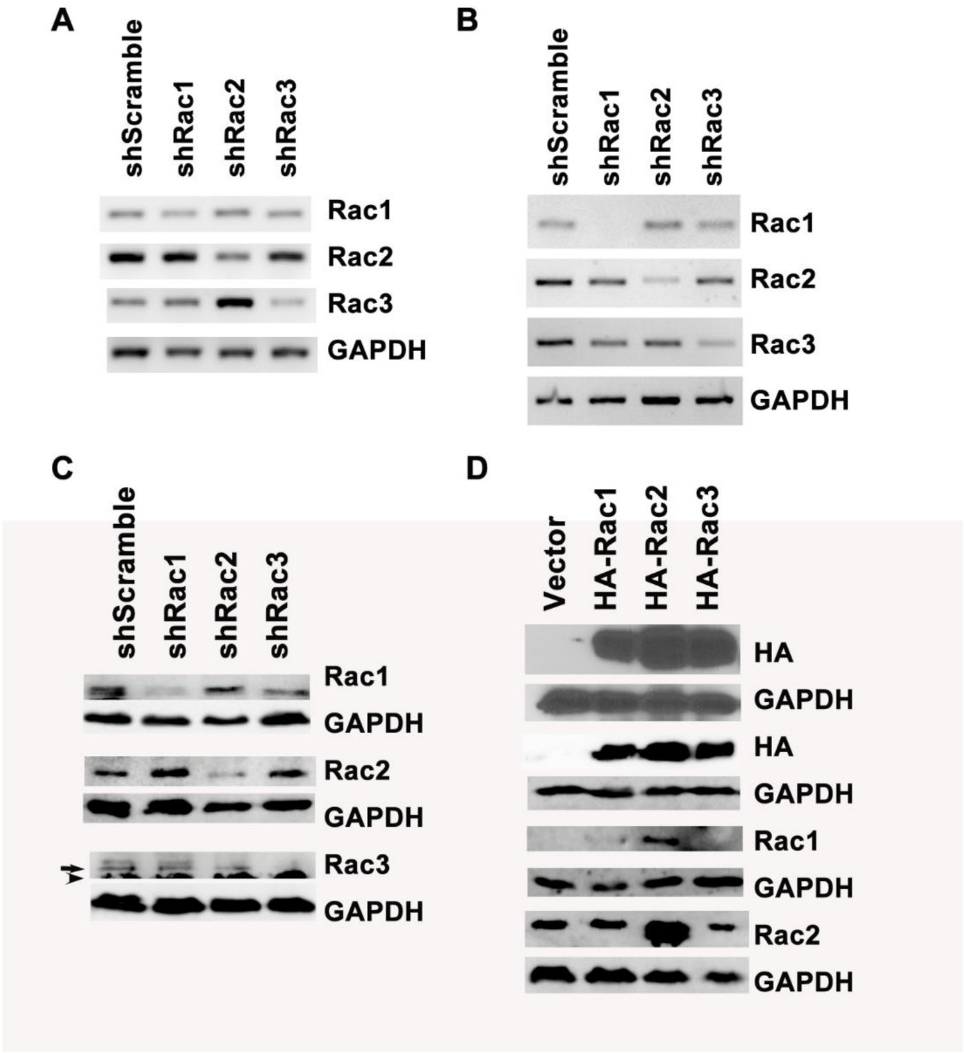

Supplementary Figure 1: The expression of Rac1-3 in Rac knocked-down cells and Rac-overexpressed cells. A. U251-tumorsphere and **B.** U373-tumorsphere harboring scramble sequences or short hairpin sequences targeting Rac1-3 were subjected to RNA extraction and RT-PCR analysis. **C.** Western blot analysis of shRac knockdown effects in U373-tumorsphere. (Arrow indicates Rac 3 and arrowhead indicates an equally-expressed non-specific band). **D.** Western blot analysis of HA-Rac protein overexpression in U251-tumorsphere (top two panels) and U373-tumorsphere (3rd and 4th panels) harboring HA-tagged Rac cDNAs. 5th and 6th panels: Rac1-specific antibody was used to detect the endogenous Rac1 expression in U373 cells. Only the overexpressed form can be detected which indicates that the endogenous one is too low to be detected compared to over-expressed one. HA-Rac2 expression was too high and non-specificly detected by Rac1 antibody, too. Bottom two panels: Rac2-specific antibody detected the endogenous and overexpressed Rac2 protein.

A

Cell lines	% of GFP$^+$	Cell lines	% of GFP$^+$
Vector	85.30	shScramble	100
Rac1	95.30	shRac1	99.4
Rac2	99.10	shRac2	99.1
Rac3	98.30	shRac3	91.4

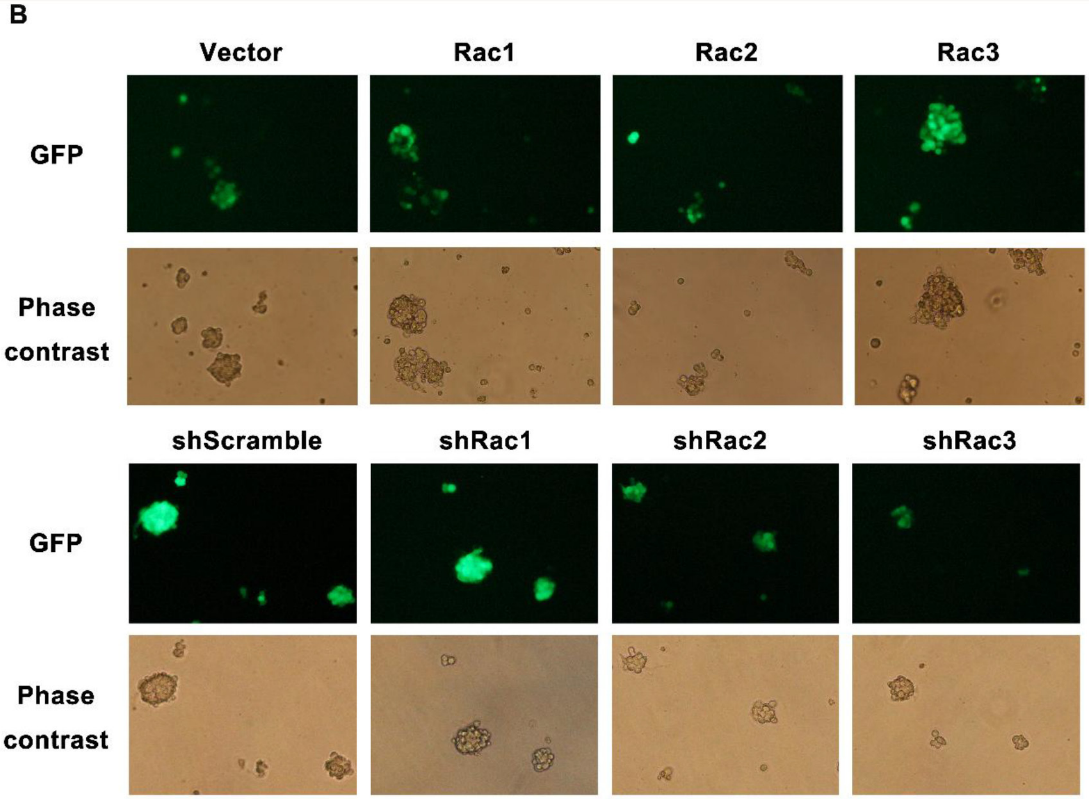

Supplementary Figure 2: The GFP expression of lentivirus stably infected cells. A. The percentage of GFP-positive cells in U373-MG cells infected by lentiviruses harboring Rac cDNAs or siRNAs were analyzed by Flow cytometry using U373-MG parental cells as control. These cells were then cultured in neurosphere culture conditions at least for one week to form tumorspheres. **B.** The spheres and the GFP expression pattern were demonstrated with images taken by inverted fluorescence microscope (DMI3000, Leica).

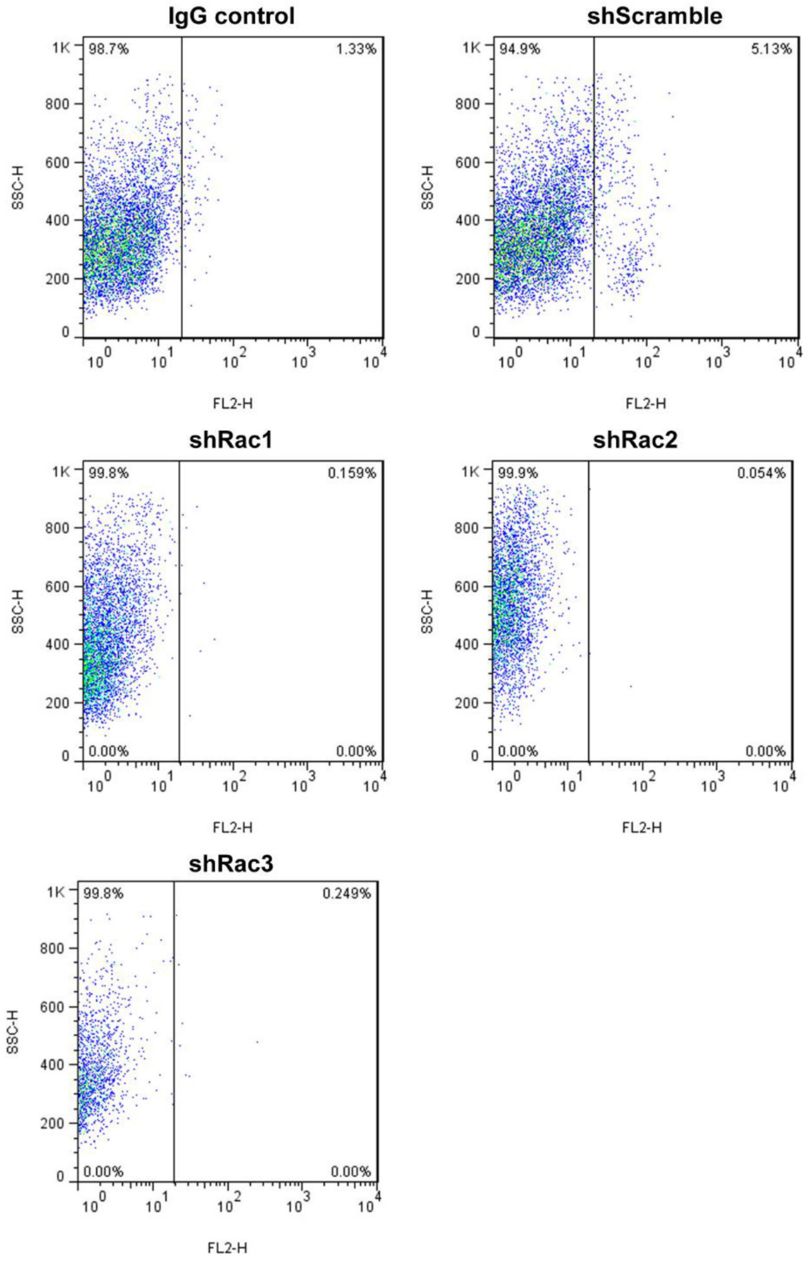

Supplementary Figure 3: The CD133+-populations analyzed by flow cytometry. U373-tumorspheres stably expressing scrambled shRNA or shRacs were incubated with PE-conjugated anti-CD133 antibody and then were subjected to flow cytometry analysis. U373-shScramble tumorspheroid cells incubated with comparable PE-conjugated IgG served as a negative control. shRacs reduced the CD133-positive cell population.

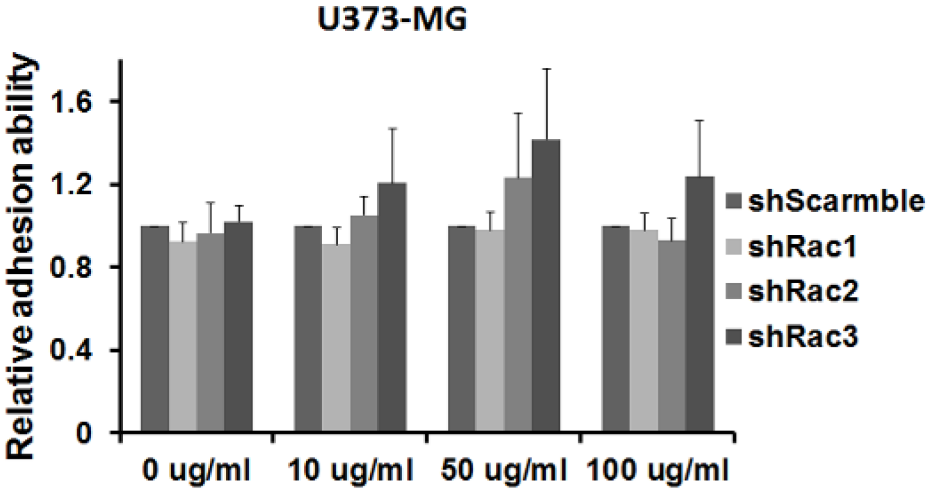

Supplementary Figure 4: Comparison of the adhesion ability of cells harboring shRacs. Ten thousand U373-MG cells stably expressing scrambled shRNA or shRacs were seeded in 96-well plate coated with different concentrations of collagen. After one hour incubation, non-adhered cells were washed away and the adhered cells were stained with 0.5% crystal violet for 15 minutes. The adhesion levels were determined by the O.D. 560 nm of the dye eluted from the adhered cells.
